# Supplementary material for: Direct and Base Excision Repair-Mediated Regulation of a GC-Rich cis-Element in Response to 5-Formylcytosine and 5-Carboxycytosine
Source: Int J Mol Sci. 2021 Oct 13;22(20):11025. doi: 10.3390/ijms222011025 (PMC8539351; doi:10.3390/ijms222011025)
Supplement: Supplementary file 1 [file ijms-22-11025-s001.zip › ijms-1391943-supplementary.pdf]

International Journal of
Molecular Sciences

MDPI

Supplementary Data

Direct and Base Excision Repair-Mediated Regulation of a GC-Rich *cis*-Element in Response to 5-Formylcytosine and 5-Carboxycytosine

Nadine Müller, Eveliina Ponkkonen, Thomas Carell, and Andriy Khobta

Supplementary Figures S1–S4

Figure S1: Effects of 5-mC, 5-hmC, 5-fC, and 5-caC on the GC box activity (representative flow cytometry experiments)

Figure S2: PCR screening for the rearranged *TDG* gene in CRISPR-Cas9-edited HeLa cells

Figure S3: Effects of BER-resistant analogs of 5-fC and 5-caC on the GC box activity in the TDG knockout cell line (clone F3)

Figure S4: Incorporation of synthetic oligonucleotides containing F and SF into the specified positions (*) in the purine-rich (a) and pyrimidine-rich (b) GC box strands.

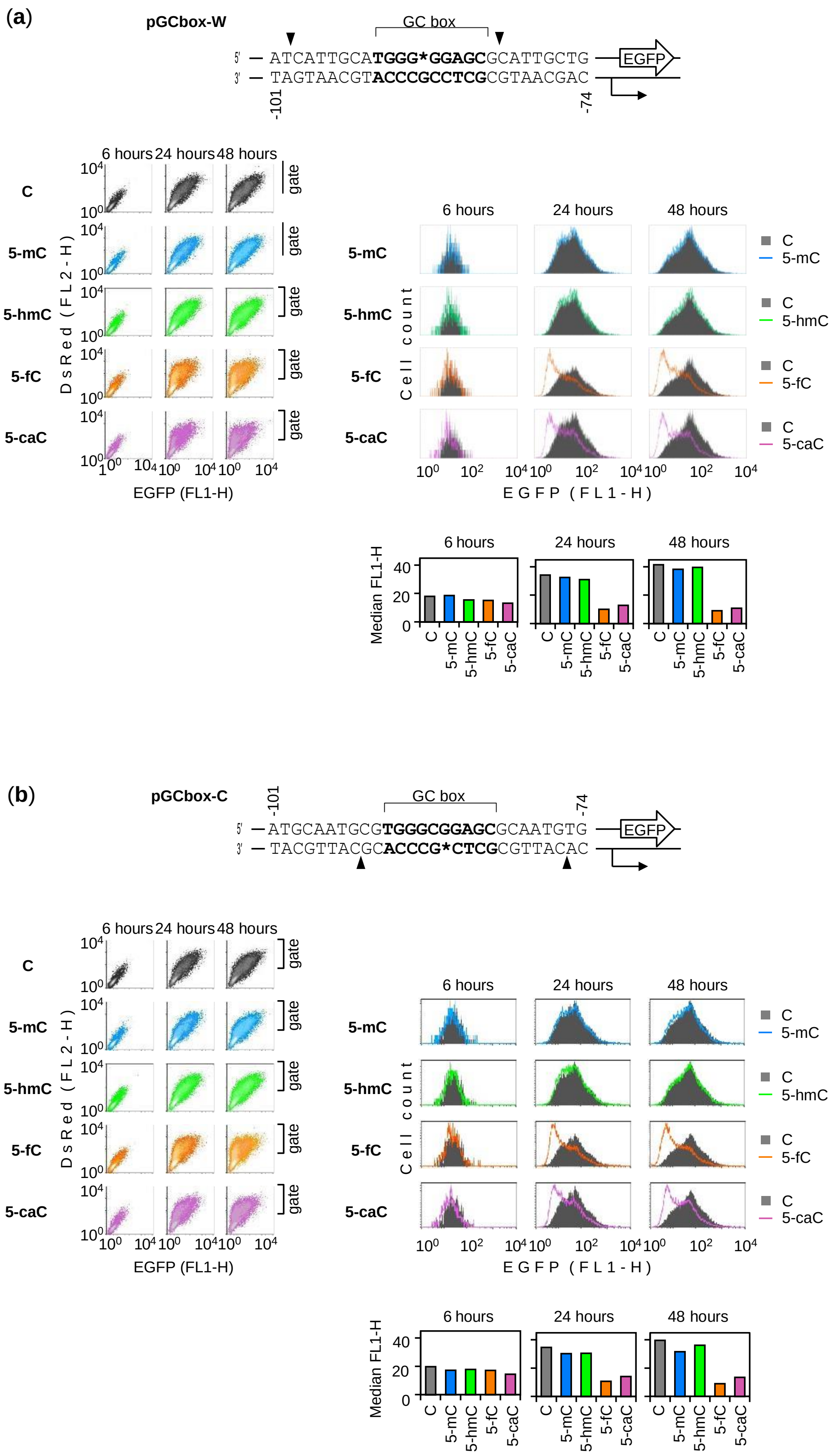

Figure S1. Effects of 5-mC, 5-hmC, 5-fC, and 5-caC on the GC box activity (representative flow cytometry experiments). Dual color fluorescence scatter-plots with gate indicated, the derived EGFP fluorescence distribution plots, and quantification of the median EGFP signal of constructs 5-mC, 5-hmC, 5-fC, and 5 caC (*): (**a**) in the purine-rich strand; (**b**) in the pyrimidine-rich strand.

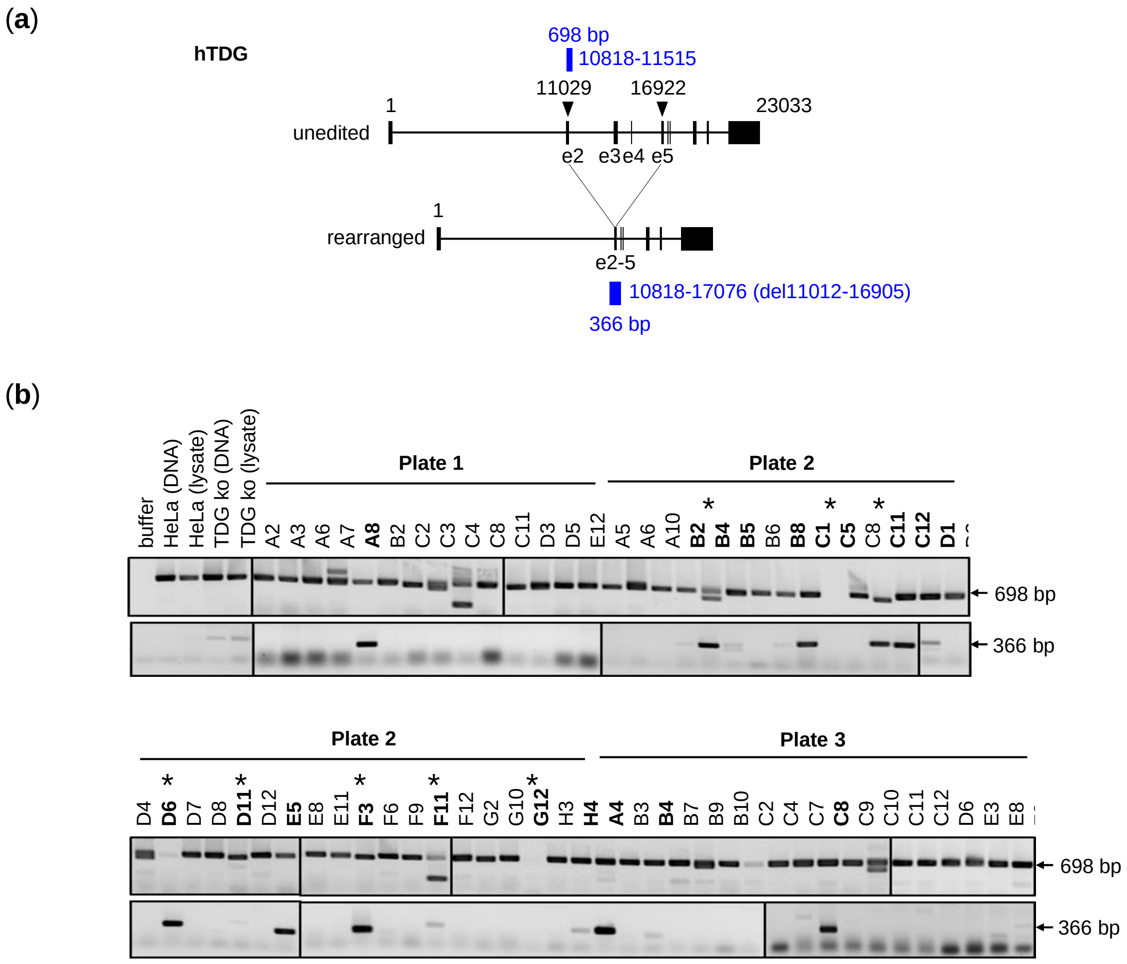

Figure S2. PCR screening for the rearranged *TDG* gene in CRISPR-Cas9-edited HeLa cells: (**a**) Scheme of targeted TDG locus in HeLa cells. The expected Cas9 cut sites are indicated with arrowheads. PCR primer positions along with the expected products (boxes) and amplicon sizes (numbers) are indicated in blue color; (**b**) PCR screening results of single clones. Marked are clones displaying the expected rearranged band (bold typeface) and those selected for the protein expression analyses (*), as shown in Figure 5B.

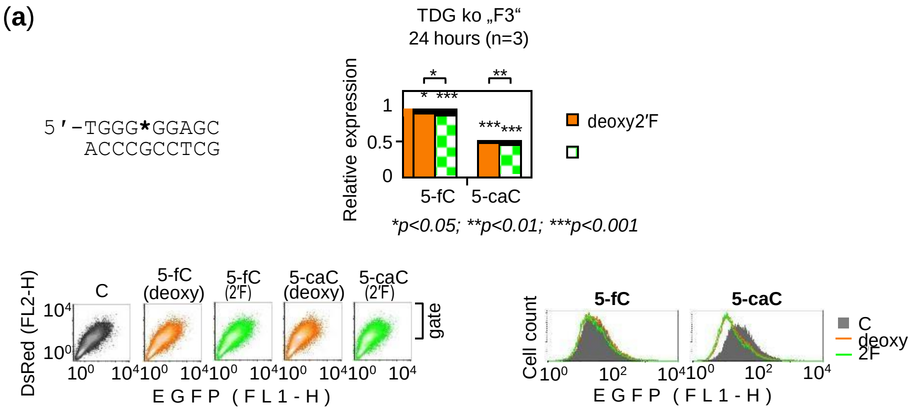

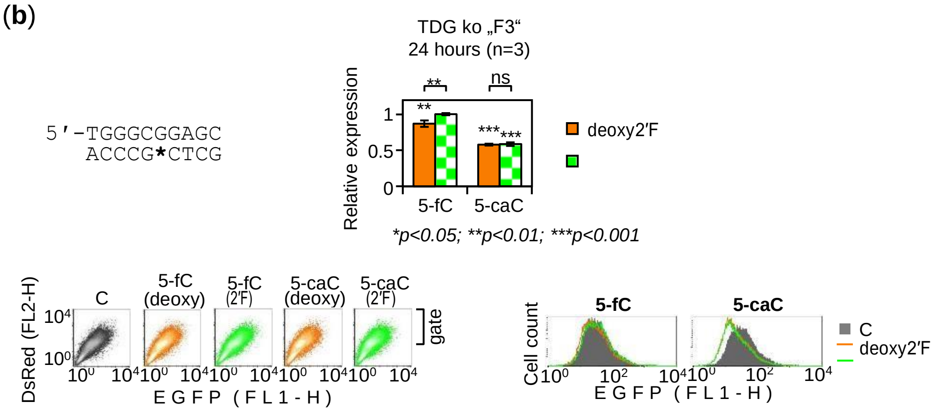

Figure S3. Effects of BER-resistant analogs of 5-fC and 5-caC on the GC box activity in the TDG knockout cell line (clone F3). The modifications were present at the indicated positions (*): (**a**) in the purine-rich GC box strand; (**b**) in the pyrimidine-rich strand.

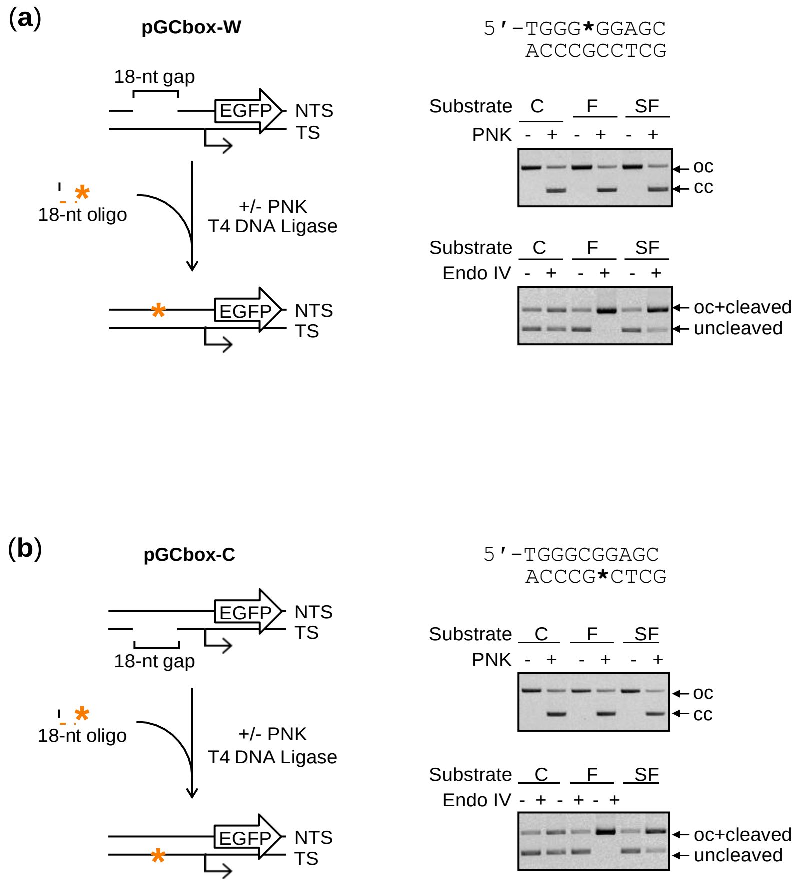

Figure S4. Incorporation of synthetic oligonucleotides containing F and SF into the specified positions (*) in the purine-rich (**a**) and pyrimidine-rich (**b**) GC box strands. Gels show the ligation controls with and without PNK and the results of incubation with endonuclease IV (Endo IV). It can be appreciated, that both lesions were incorporated with high efficiencies (upper gels). The incision by endonuclease IV is efficient at F, but largely inhibited at SF (lower gels).
